# Histone acetyltransferase-deficient p300 mutants in diffuse large B cell lymphoma have altered transcriptional regulatory activities and are required for optimal cell growth

**DOI:** 10.1186/1476-4598-13-29

**Published:** 2014-02-15

**Authors:** Leila Haery, Julián G Lugo-Picó, Ryan A Henry, Andrew J Andrews, Thomas D Gilmore

**Affiliations:** 1 Department of Biology, Boston University, 5 Cummington Mall, Boston, MA 02215, USA; 2 Fox Chase Cancer Center, Philadelphia, PA 19111, USA

**Keywords:** p300, B-cell lymphoma, Histone acetyltransferase, SUDHL2, REL, H3 acetylation

## Abstract

**Background:**

Recent genome-wide studies have shown that approximately 30% of diffuse large B-cell lymphoma (DLBCL) cases harbor mutations in the histone acetyltransferase (HAT) coactivators p300 or CBP. The majority of these mutations reduce or eliminate the catalytic HAT activity. We previously demonstrated that the human DLBCL cell line RC-K8 expresses a C-terminally truncated, HAT-defective p300 protein (p300ΔC-1087), whose expression is essential for cell proliferation.

**Methods:**

Using results from large-scale DLBCL studies, we have identified and characterized a second C-terminally truncated, HAT-defective p300 mutant, p300ΔC-820, expressed in the SUDHL2 DLBCL cell line. Properties of p300ΔC-820 were characterized in the SUDHL2 DLBCL cell line by Western blotting, co-immunoprecipitation, and shRNA gene knockdown, as well by using cDNA expression vectors for p300ΔC-820 in pull-down assays, transcriptional reporter assays, and immunofluorescence experiments. A mass spectrometry-based method was used to compare the histone acetylation profile of DLBCL cell lines expressing various levels of wild-type p300.

**Results:**

We show that the SUDHL2 cell line expresses a C-terminally truncated, HAT-defective form of p300 (p300ΔC-820), but no wild-type p300. The p300ΔC-820 protein has a wild-type ability to localize to subnuclear “speckles,” but has a reduced ability to enhance transactivation by transcription factor REL. Knockdown of p300ΔC-820 in SUDHL2 cells reduced their proliferation and soft agar colony-forming ability. In RC-K8 cells, knockdown of p300ΔC-1087 resulted in increased expression of mRNA and protein for REL target genes A20 and IκBα, two genes that have been shown to limit the growth of RC-K8 cells when overexpressed. Among a panel of B-lymphoma cell lines, low-level expression of full-length p300 protein, which is characteristic of the SUDHL2 and RC-K8 cells, was associated with decreased acetylation of histone H3 at lysines 14 and 18.

**Conclusions:**

The high prevalence of p300 mutations in DLBCL suggests that HAT-deficient p300 activity defines a subtype of DLBCL, which we have investigated using human DLBCL cell lines RC-K8 and SUDHL2. Our results suggest that truncated p300 proteins contribute to DLBCL cell growth by affecting the expression of specific genes, perhaps through a mechanism that involves alterations in global histone acetylation.

## Introduction

Diffuse large B-cell lymphoma (DLBCL) accounts for approximately 30% of B-cell lymphoma cases [1]. Molecular profiling of DLBCL cell lines and patient tumors has led to the identification of distinct subtypes, which has been a useful tool in predicting patient survival and therapeutic response [2]. Genome-wide studies have shown that approximately 30% of DLBCL tumors harbor mutations in two highly related histone acetyltransferase (HAT) genes, *EP300* and *CREBBP*[3-7].

*EP300* and *CREBBP* encode related HATs, p300 and CBP, respectively, that have widespread genomic effects on chromatin structure and gene expression as well as non-genomic effects on protein function [8]. These HATs serve as coactivators for many transcription factors, either through acetylation of lysine residues on histones to modify DNA structure at sites of active transcription or through acetylation of transcription factors to modify their activity. In both cases, the centrally-located, catalytic HAT domain is required for these effects on transcription. Consistent with its broad role in transcriptional control, p300 can directly interact with a wide variety of transcription factors, including NF-κB [9,10], p53 [11,12], MyoD [13], HIF-1α [14], BRCA1 [15], and Ets-1 [16]. In addition, p300 and CBP contain several protein-protein interaction domains and can exhibit HAT-independent functions; for example, p300 can enhance transcription simply by recruiting proteins to transcriptional start sites, including members of the transcription pre-initiation complex and the RNA polymerase holo-enzyme [8,17].

Most p300/CBP mutations identified in DLBCL are point mutations, nonsense mutations, or deletions that disable HAT activity [3,5,10,18]. In some epithelial cancers where a truncated p300/CBP protein is expressed, the wild-type allele is silenced or otherwise inactivated [19], and ectopic expression of wild-type p300 in some HAT-deficient p300 cancer cell lines slows cell growth [7,20]. Such results have led p300 to be classified as a tumor suppressor, arising from the hypothesis that it is the loss of wild-type p300 activity which contributes to oncogenesis.

We have previously shown that, due to a 3’ alteration in one copy of the *EP300* gene, the DLBCL cell line RC-K8 expresses a C-terminally truncated HAT-deficient p300 protein (herein called p300ΔC-1087). Even though the other copy of the *EP300* locus appears intact, RC-K8 cells express low to undetectable levels of wild-type p300 mRNA and protein [10,18]. We previously reported that the RC-K8 p300ΔC-1087 could not act as a coactivator for the REL transcription factor [18]. Of note, knockdown of p300ΔC-1087 expression reduces the proliferation and soft agar colony-forming ability of RC-K8 cells [18], and re-expression of wild-type p300 is tolerated in RC-K8 cells, but sensitizes them to the cell killing effects of small-molecule BCL6 inhibitors [7]. Other studies have demonstrated that expression of a HAT domain mutant of p300 results in increased proliferation of hematopoietic stem and progenitor cells, whereas complete loss of p300 does not [21]. Such findings suggest that p300 HAT activity normally limits B-cell proliferation, and that expression of p300 proteins with an inactive catalytic domain contributes to B-cell growth, survival, and tumorigenesis.

In this report, we have characterized a truncated p300 protein expressed in the DLBCL cell line SUDHL2. We show that this C-terminally truncated and HAT-deficient p300 mutant is a weak transcriptional coactivator, and that its expression is required for the optimal growth of SUDHL2 cells. These results and others suggest that expression of C-terminally truncated p300 coactivators defines a subset of DLBCL that utilize distinct oncogenic pathways.

## Results

### The SUDHL2 DLBCL cell line expresses a C-terminally truncated p300 protein

**Figure 1:**
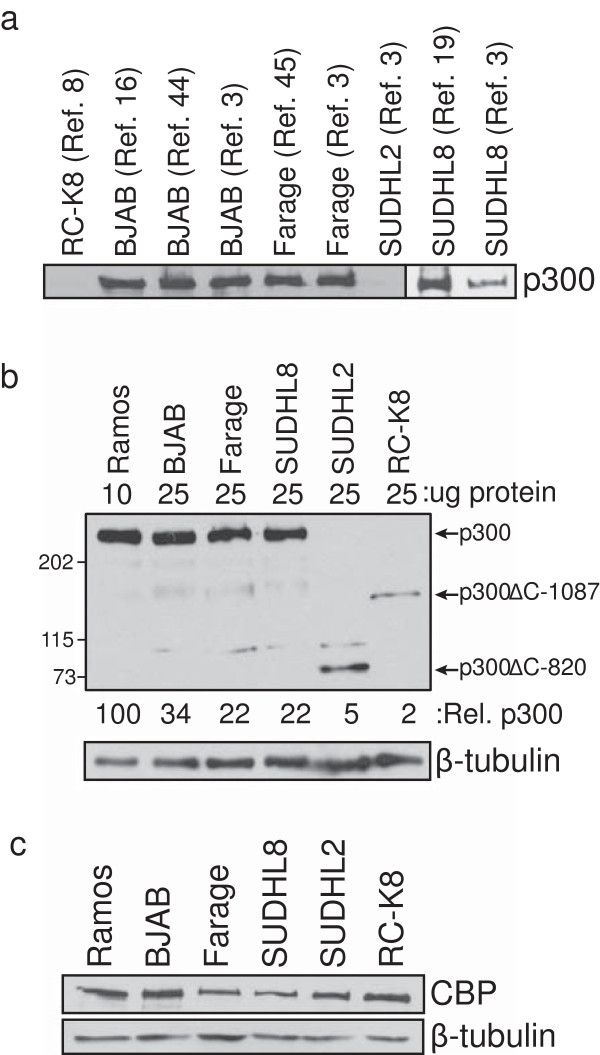
**Expression of p300 proteins in DLBCL cell lines. (a)** Anti-p300 Western blotting was performed on whole-cell extracts from the indicated DLBCL cell lines. The sources of the cell lines are described in Materials and methods and in the indicated references in the figure. Thirty-five μg of total protein from whole-cell extracts was loaded for all samples except for RC-K8 (50 μg). **(b)** SUDHL2 cells express a C-terminally truncated p300 protein (p300ΔC-820). Whole-cell extracts from the six indicated cell lines were analyzed by Western blotting for relative expression of p300. The amount of total cell protein loaded is indicated. Western blots were performed with N-terminal anti-p300 antiserum. p300 expression was quantified by densitometric analysis and is relative to full-length p300 in Ramos cells (100). Relative p300 expression for the SUDHL2 and RC-K8 cell lines was determined by analyzing the bands corresponding to p300ΔC-820 and p300ΔC-1087, respectively. Full-length p300 in SUDHL2 and RC-K8 cell lines was undetectable (0). **(c)** Whole-cell extracts from the six indicated DLBCL cell lines were analyzed by Western blotting for expression of CBP. Twenty-five μg of whole-cell extract was used for each sample. Western blotting was performed with anti-CBP and anti-β-tubulin (as a loading control) antisera.

Pasqualucci *et al*. [3] reported that several human DLBCL cell lines, including BJAB, Farage, SUDHL2 and SUDHL8, express no detectable full-length p300 protein. Because of our ongoing interest in p300 mutations in DLBCL [10,18], we sought to further characterize p300 status in these four cell lines. In particular, the reported absence of full-length p300 in BJAB and Farage cells is in contrast to our previous findings [10] and those of others [7,22-25]. Therefore, we reassessed p300 expression in these four DLBCL cell lines by Western blotting of whole-cell extracts, and included multiple independent cell lines in our analyses (including the ones used by Pasqualucci *et al*. [3]). As a further control, we analyzed RC-K8 cells, which we have previously shown express little or no full-length p300 [10,18]. As shown in Figure 1a, multiple isolates of BJAB, Farage, and SUDHL8 cells express easily detectable levels of full-length p300. In contrast, RC-K8 and SUDHL2 cells do not express detectable levels of full-length p300.

Although full-length p300 is not expressed in RC-K8 cells, these cells do express a C-terminally truncated p300 protein (p300ΔC-1087) [10,18]. Due to the absence of full-length p300 in SUDHL2 cells (Figure 1a) and the reported nonsense mutation at *EP300* codon 821 in these cells [3], we next determined whether SUDHL2 cells express a C-terminally truncated form of p300. Western blotting using an antibody against N-terminal sequences of p300 showed that SUDHL2 cells express a smaller form of p300 under conditions where there is no detectable full-length p300 (Figure 1b). Consistent with the size expected for the reported codon 821 nonsense mutation [3], the single anti-p300-reactive protein in SUDHL2 cell lysates migrated at approximately 90 kDa (herein called p300ΔC-820).

Pasqualucci *et al*. [3] reported that SUDHL2 cells are hemizygous for the *EP300* gene and contain only the codon 821 mutant allele. To confirm that SUDHL2 cells lack wild-type *EP300* sequences, we PCR-amplified and then sequenced *EP300* genomic DNA flanking codon 821 in exon 14. Our results confirmed that the amplified DNA only contained the C2856T mutation (CAG to TAG) at codon 821 in exon 14 (Additional file 1), demonstrating that there is no wild-type *EP300* locus in SUDHL2 cells.

Quantitation of p300 levels indicated that the truncated p300 proteins in RC-K8 and SUDHL2 cells are expressed at low levels compared to full-length p300 in four other B-lymphoma cell lines (Figure 1b, relative p300 values). In contrast, the levels of the related HAT CBP were similar in all six cell lines, and certainly did not vary depending on whether a given cell line expressed full-length or truncated p300 (Figure 1c). These results demonstrate that neither overexpression of mutant p300 nor compensatory changes in CBP expression occurs in B-lymphoma cell lines expressing truncated p300 mutants. Instead, the expression of a truncated p300 appears to be associated with reduced expression of full-length p300.

### Characterization of the p300ΔC-820 protein from SUDHL2 cells

**Figure 2:**
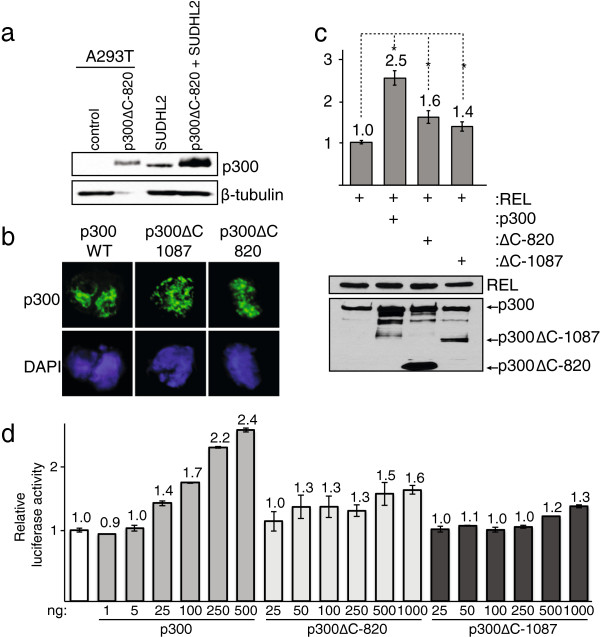
**p300ΔC**-**820 localizes to the nucleus**, **but only weakly enhances REL transactivation. (a)** Anti-p300 Western blotting was performed on extracts from control A293T cells, p300ΔC-820-transfected A293T cells, and SUDHL2 cells. Twenty-five μg of control A293T and SUDHL2 total protein and 30 ng of p300ΔC-820-transfected A293T total protein were loaded. p300ΔC-820-transfected A293T and SUDHL2 extracts were loaded together in the last lane. **(b)** DF-1 chicken fibroblasts were transfected with 3 μg of pCMVβ-p300, pCMVβ- p300ΔC-1087, or pCMVβ- p300ΔC-820 as indicated. Indirect immunofluorescence was performed using a primary anti-p300 antiserum and a FITC-conjugated anti-rabbit secondary antibody (top row). Nuclei were visualized by DAPI staining (bottom row). **(c)** A293 cells were co-transfected with 0.5 μg of pcDNA-REL along with 0.5 μg of pCMVβ-p300, pCMVβ-p300ΔC-1087, or pCMVβ-p300ΔC-820 as indicated. Western blotting shows relative p300 and REL expression in transfected cells (bottom panel). Luciferase and β-galactosidase activities were determined, and values were normalized to control transfections (1.0, top panel). Error bars represent standard error of the mean. Asterisks represent p-value < 0.05 for the difference between the indicated value and the control value (1.0) in a one-tailed t-test. **(d)** A293 cells were co-transfected with 0.5 μg of pcDNA-REL and the indicated amounts of pCMVβ-p300, pCMVβ-p300ΔC-820, or pCMVβ-p300ΔC-1087. Luciferase and β-galactosidase activities were determined, and values were normalized to REL-alone control transfections as indicated (1.0, white bar). Total DNA within each experiment was kept constant by the addition of empty pcDNA3.1 vector. For all reporter assays, values are the averages of at least three experiments, each performed with duplicate or triplicate samples. Error bars represent standard error of the mean.

To further characterize the p300ΔC-820 protein, we created a CMV promoter-based plasmid expression vector for p300ΔC-820 by mutating codon 821 to a stop codon in a full-length p300 cDNA, based on the nonsense mutation in SUDHL2 genomic DNA. When transfected into A293T cells, the synthetic p300ΔC-820 cDNA directed the expression of a protein that nearly co-migrated with p300ΔC-820 from SUDHL2 cells (Figure 2a).

We next analyzed the subcellular localization of ectopically expressed p300ΔC-820 in transfected fibroblasts. As shown in Figure 2b, overexpressed wild-type p300, p300ΔC-1087, and p300ΔC-820 predominantly localized to discrete punctate regions of the nucleus (“speckles”) in transfected fibroblasts. This speckled localization of p300ΔC-820 is similar to the staining seen with wild-type p300 and p300ΔC-1087 (from RC-K8 cells), but is distinct from the overall nuclear DNA staining seen with DAPI.

We have previously shown that wild-type p300 can enhance the ability of transcription factor REL to activate a multimeric κB-site reporter gene, whereas p300ΔC-1087 cannot [18]. In more extensive studies, we now find that high amounts of p300ΔC-820 and p300ΔC-1087 can weakly enhance transactivation by REL. Consistent with previous results [18], wild-type p300 enhanced the ability of REL to activate the κB-site reporter in A293 cells by approximately 2.5-fold (Figure 2c). In contrast, p300ΔC-820 and p300ΔC-1087 enhanced REL-dependent transactivation, on average, by only 1.6- and 1.4-fold, respectively (Figure 2c). To further assess the ability of these C-terminally truncated mutants to enhance REL-dependent transactivation, we co-transfected a constant amount of REL plasmid with increasing amounts of either p300ΔC-820 or p300ΔC-1087 expression plasmids and measured transcriptional activation of the κB-site reporter. Like wild-type p300, both p300ΔC-820 and p300ΔC-1087 enhanced REL-dependent transactivation in a generally dose-dependent manner (Figure 2d). However, both p300 mutants enhanced transcription to a lesser extent than an equimolar amount of wild-type p300 (e.g., 500 ng of p300ΔC-820 plasmid enhanced REL-dependent transactivation by 1.5-fold, whereas 500 ng of wild-type p300 plasmid resulted in a 2.4-fold enhancement). Together, these results demonstrate that both p300ΔC-820 and p300ΔC-1087 can enter the nucleus and function as relatively weak coactivators for REL-dependent transactivation.

### p300ΔC-820 interacts with REL transactivation domain sequences *in vitro* and *in vivo*

**Figure 3:**
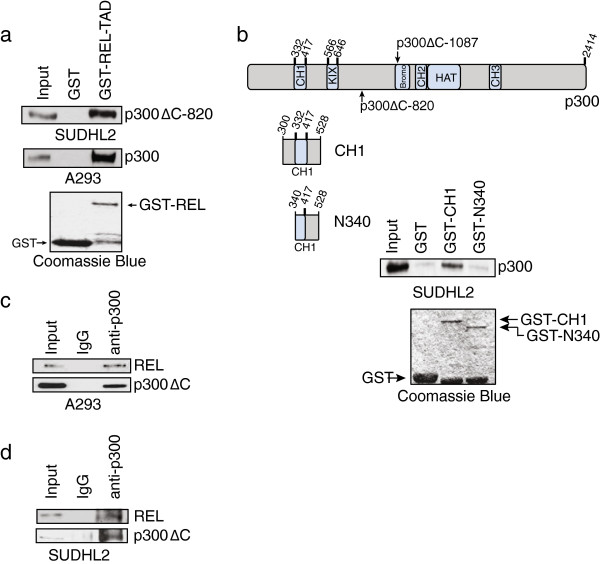
**p300ΔC**-**820 interacts with REL *in vitro *and *in vivo*. (a)** A GST-REL-TAD (aa 324-587) pulldown was performed on whole-cell extracts from SUDHL2 (p300ΔC-820, top panel) or A293 cells (wild-type 300, middle panel). Bound proteins were subjected to anti-p300 Western blotting to detect p300ΔC-820 or WT p300, as indicated. **(b)** GST-p300 pulldown of endogenous REL from SUDHL2 whole-cell extracts. Bound proteins were subjected to anti-REL Western blotting. The structure of wild-type p300 is shown and structures of GST-p300 mutants used are indicated. **(c)** Nuclear extracts of A293 cells co-transfected with p300ΔC-820 and REL were immunoprecipitated with normal rabbit IgG or anti-p300 antiserum. Immunoprecipitates were subjected to anti-REL or anti-p300 Western blotting, as indicated. One percent (3 μg) of the nuclear extract used for immunoprecipitation was included as an input for the anti-REL blot and 10% (30 μg) of the nuclear extract was used as input for the anti-p300 blot. **(d)** Nuclear extracts of SUDHL2 cells were immunoprecipitation with normal rabbit IgG or anti-p300 antiserum. Immunoprecipitates were subjected to anti-REL or anti-p300 Western blotting, as indicated. Ten percent (25 μg) of the nuclear extract used for immunoprecipitate was included as an input for the anti-REL blot and 30% (75 μg) of the nuclear extract was used as input for the anti-p300 blot. Coomassie Blue staining was performed on 5% of GST or GST-fusion protein used in the pulldowns (**a**, **b** lower panels).

We have previously shown that p300ΔC-1087 retains the ability to interact with transcription factor REL *in vitro* and in RC-K8 cells, and that this interaction primarily occurs via the C-terminal transactivation domain (TAD) of REL [10]. To determine whether p300ΔC-820 also has the ability to interact with REL, a pull-down assay was performed using a GST-REL-TAD fusion protein and whole-cell extracts from SUDHL2 cells. Anti-p300 Western blotting showed that GST-REL-TAD specifically pulled down p300ΔC-820 (Figure 3a). As a control, GST-REL-TAD was incubated with whole-cell extracts from A293 cells, where it was able to pull down endogenous wild-type p300 protein (Figure 3a). Moreover, a GST-p300 fusion protein containing the CH1 protein interaction domain (aa 300-528, retained in p300ΔC-820) could pull-down REL from SUDHL2 whole-cell extracts (Figure 3b). This ability to pull-down REL was lost when a smaller CH1 region was used (aa 340-528) (Figure 3b), indicating that the entire CH1 domain of p300 is required for efficient binding to REL.

To determine whether p300ΔC-820 retains the ability to interact with REL *in vivo*, we performed an anti-p300 immunoprecipitation from A293 cells co-transfected with expression plasmids for p300ΔC-820 and REL. Anti-REL Western blotting of an anti-p300 immunoprecipitate demonstrated that REL can interact with p300ΔC-820, at least when overexpressed in a non-lymphoid cell type (Figure 3c). To determine whether endogenous p300ΔC-820 and REL interact in SUDHL2 cells, we performed an anti-p300 immunoprecipitation on nuclear extracts. Anti-REL Western blotting of the anti-p300 immunoprecipitate demonstrated that REL is also present, indicating that REL and p300ΔC-820 interact in SUDHL2 cells (Figure 3d).

### Knockdown of p300ΔC-820 reduces the growth of SUDHL2 cells

**Figure 4:**
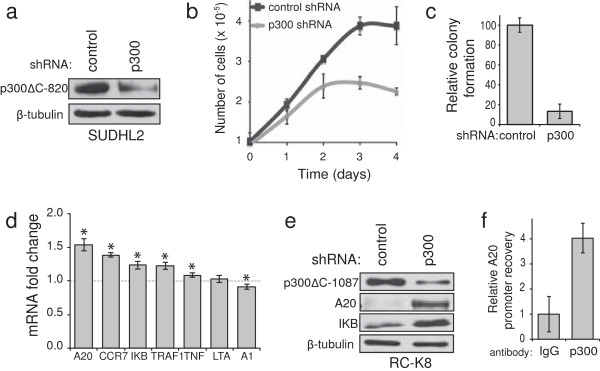
**Knockdown of p300ΔC reduces proliferation of SUDHL2 cells and increases A20 and IκBα expression in RC**-**K8 cells. (a)** Anti-p300 Western blotting was performed on whole-cell extracts from SUDHL2 cells expressing p300 shRNA or control shRNA. Anti-β-tubulin Western blotting is a loading control. **(b)** 10^5^ SUDHL2 cells expressing p300 shRNA or control shRNA were plated in 500 μl RPMI/10% FBS in 16-mm wells. On each day, three wells of each cell type were counted. **(c)** Soft agar colony assays were performed using SUDHL2 cells expressing p300 shRNA or control shRNA. Results are averages of three experiments performed with triplicate plates containing 2000 or 5000 cells. Colony numbers are normalized to the number of colonies formed by SUDHL2 cells expressing control shRNA (100). Error bars represent standard deviation. **(d)** Reverse transcribed total RNA from RC-K8 cells expressing p300 shRNA or a control shRNA was subjected to real-time qPCR using gene-specific primers. mRNA expression values from RC-K8 cells expressing p300 shRNA were compared to values from RC-K8 cells expressing control shRNA to determine the fold change in expression. Error bars represent standard error of the mean. Asterisks indicate p-values < 0.05 for the difference in mRNA levels relative to control cells (=1.0) using a two-tailed t test. **(e)** Anti-p300, anti-A20, and anti-IκBα Western blotting was performed on whole-cell extracts from RC-K8 cells expressing p300 shRNA or control shRNA. Anti-β-tubulin Western blotting is a loading control. **(f)** RC-K8 cells were subjected to crosslinking with formaldehyde and extracts from isolated nuclei were then immunoprecipitated using normal rabbit IgG or anti-p300 antiserum. The crosslinks were reversed and DNA in immunoprecipitates was subjected to qPCR using primers spanning the *A20* promoter region. Relative *A20* promoter PCR product was normalized to the IgG control (1.0). Error bars represent the standard error of the mean.

To determine whether p300ΔC-820 contributes to the growth of SUDHL2 cells (which contain no wild-type *EP300* allele), we first knocked down expression of p300ΔC-820 in these cells with a retroviral vector containing a short hairpin RNA (shRNA) that has been previously shown to knock down expression of wild-type p300 [26] and p300ΔC-1087 [18]. Western blotting showed that p300ΔC-820 expression was reduced by approximately 67% in a pool of SUDHL2 cells expressing p300 shRNA as compared to SUDHL2 cells expressing a control, non-targeting shRNA (Figure 4a). We next compared the proliferation of SUDHL2 cells expressing p300 shRNA and control shRNA by counting cells over the course of four days. Knockdown of p300ΔC-820 reduced the proliferation of SUDHL2 in liquid medium (Figure 4b). In addition, SUDHL2 cells with reduced expression of p300ΔC-820 formed approximately 8-fold fewer colonies in soft agar than control SUDHL2 cells (Figure 4c). Thus, p300ΔC-820 appears to contribute to *in vitro* growth properties of SUDHL2 cells.

### p300ΔC-1087 suppresses the expression of NF-κB-regulated genes encoding A20 and IκBα

Previous results have shown that several REL/NF-κB target genes are highly expressed in RC-K8 cells [27]. Because REL and p300ΔC-1087 interact in RC-K8 cells [10], we sought to determine whether knockdown of p300ΔC-1087 would affect expression of some known REL-regulated genes in RC-K8 cells. Therefore, qPCR was performed to compare mRNA levels of seven such genes (*A20*, *BCL2A1*, *CCR7*, *NFKBIA* [encoding IκBα], *LTA*, *TNF*, *TRAF1*) in RC-K8 cells expressing p300 shRNA to control cells (Figure 4d). First we confirmed by Western blotting that p300ΔC-1087 expression was reduced in RC-K8 cells expressing p300 shRNA as compared to RC-K8 cells expressing a control, non-targeting shRNA (Figure 4e, top panel). As shown in Figure 4d, expression of *A20*, *CCR7*, *NFKBIA*, *TRAF1*, and *TNFα* mRNAs was significantly increased (1.1- to 1.5-fold; p < 0.05) in p300 knockdown cells, relative to control RC-K8 cells. Expression of *A1* and *LTA* were not significantly increased in RC-K8 cells expressing p300 shRNA.

Extracts from RC-K8 cells with knockdown of p300ΔC-1087 were next subjected to anti-A20 and anti-IκBα Western blotting to determine whether the increases in mRNA seen in these cells resulted in increases in protein levels. As shown in Figure 4e, A20 and IκBα protein levels were increased in RC-K8 cells expressing p300 shRNA. β-tubulin expression was not affected by p300ΔC-1087 knockdown.

We then sought to determine whether the p300ΔC-1087 protein is located at the *A20* promoter in RC-K8 cells. Therefore, we performed a ChIP assay in which p300ΔC-1087 was immunoprecipitated from RC-K8 cell nuclei and, after reversing crosslinks, qPCR was performed using primers surrounding the κB sites of the *A20* promoter. As shown in Figure 4f, *A20* promoter sequences were enriched by approximately four-fold (as compared to the IgG control) in an anti-p300ΔC-1087 immonoprecipitate from RC-K8 cells.

The expression of p300ΔC-1087 is thus associated with a reduction in A20 and IκBα expression at both the mRNA and protein levels. Furthermore, p300ΔC-1087 can be found at the *A20* promoter, suggesting that p300ΔC-1087 has an inhibitory effect on the expression of *A20*. This analysis was done on RC-K8 cells, rather than SUDHL2 cells, because SUDHL2 cells express a mutant form of A20 protein that is unstable and difficult to detect [28].

### Wild-type p300 expression may contribute to overall histone H3 acetylation in B-cell lymphoma

**Figure 5:**
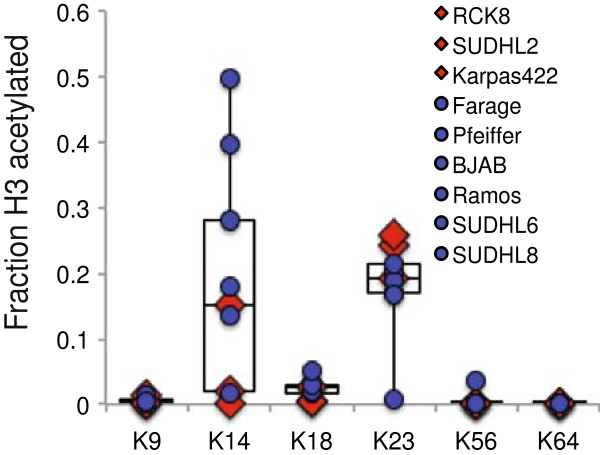
**Total histone H3 lysine acetylation profile in DLBCL cell lines.** Total histones were extracted from the indicated cell lines, were trypsinzed, and subjected to mass-spec analysis. For each H3 lysine residue, the amount of the acetylated lysine residue was measured relative to total amount of acetylated plus unmodified residue. Cell lines indicated by red diamonds express undetectable levels of wild-type p300 (RC-K8, SUDHL2) or wild-type CBP (Karpas422) by Western blotting. Cell lines indicated by blue circles express detectable levels of p300 and CBP by Western blotting. A box plot of the fraction of acetylated lysine residue out of total acetylated plus unmodified H3 was plotted for each lysine residue. For cell lines indicated by red diamonds, the mean values and 95% CIs for H3 lysine acetylation were as follows: H3K14 (0.058 ± 0.091), H3K18 (0.012 ± 0.014), and H3K23 (0.231 ± 0.039). For cell lines indicated by blue circles, the means and 95% CIs were H3K14 (0.252 ± 0.141), H3K18 (0.030 ± 0.010), and H3K23 (0.158 ± 0.061).

Because DLBCL cell lines expressing C-terminally truncated, HAT-deficient p300 proteins do not express detectable amounts of wild-type p300, we next asked whether there was any reduction in overall histone acetylation in cell lines lacking wild-type p300 among a small panel of B-lymphoma cell lines. To do this, we isolated histones from six B-lymphoma cell lines that express varying levels of wild-type p300, from RC-K8 and SUDHL2 (which express no detectable wild-type p300 protein), and from Karpas422 cells (which express detectable wild-type p300 but no detectable CBP [5] [data not shown]), and then used a mass spectrometry-based approach to quantify the degree of acetylation of six lysine residues on histone H3. The value obtained for each lysine residue represents the fraction of that residue that was acetylated relative to the total amount of each residue that was acetylated plus unmodified (Figure 5, Additional files 2 and 3). Acetylation of H3K9, K56, and K64 was low and sometimes undetectable in all nine cell lines. In contrast, acetylation of H3K14, K18, and K23 was within a detectable range and varied among the different cell lines. Cell lines with reduced wild-type p300 or CBP (i.e., RC-K8, SUDHL2, and Karpas422, indicated by red diamonds) had average or below average levels of H3K14 and H3K18 acetylation (mean and 95% CIs were H3K14 (0.058 ± 0.091) and H3K18 (0.012 ± 0.014), as compared to the broad range of H3K14 and H3K18 acetylation values found in cell lines with detectable levels of full-length p300 and CBP (H3K14, mean 0.252 ± 0.141, H3K18, mean 0.030 ± 0.010) (i.e., Farage, Pfeiffer, BJAB, Ramos, SUDHL6, and SUDHL8, indicated by blue circles) (Figure 5, Additional file 2). In contrast, cell lines lacking detectable wild-type p300 or CBP (i.e., SUDHL2, RC-K8, and Karpas422) did not have below average levels of H3K23 acetylation (mean 0.231 ± 0.039), relative to the broad range of acetylation values exhibited by cell lines with detectable levels of full-length p300 and CBP (mean 0.158 ± 0.061).

## Discussion

In this report, we have characterized molecular properties of the HAT-deficient p300ΔC-820 protein from the human DLBCL cell line SUDHL2. This is only the second truncated p300 mutant that has been functionally characterized in a human DLBCL cell line [10,18]. We show that p300ΔC-820 is the only form of p300 protein expressed in SUDHL2 cells and that p300ΔC-820 contributes to SUDHL2 cell growth, as knockdown of p300ΔC-820 expression compromised the liquid media and soft agar growth of SUDHL2 cells. Like wild-type p300, p300ΔC-820 localizes to the nucleus and can interact with NF-κB family member REL, but p300ΔC-820 has a reduced ability to enhance REL-dependent transactivation in reporter assays. As such, p300ΔC mutants have the potential to attenuate expression of transcription factor-specific target genes by preventing the interaction of transcription factors with other functionally intact coactivators. Indeed, knockdown of p300ΔC-1087 in RC-K8 cells resulted in increased expression of NF-κB target genes *A20*, *CCR7*, *NFKBIA*, *TRAF1* and *TNFα*, as well as an increase in A20 and IκBα protein expression. Finally, the RC-K8 and SUDHL2 cell lines, which have reduced expression of wild-type p300, had generally reduced levels of acetylation of histone H3 K14 and K18 among a panel of B-lymphoma cell lines.

Like wild-type p300 and the p300ΔC-1087 protein from RC-K8 cells, p300ΔC-820 showed a punctate pattern of nuclear staining by immunofluorescence, which has been associated with sites of active transcription for wild-type p300 [29]. Using reporter assays, p300ΔC-820 and p300ΔC-1087 are both weak transcriptional coactivators for REL in A293 cells (Figures 2c,d). Because p300 acts as a transcriptional coactivator through both HAT-dependent and HAT-independent mechanisms, the limited coactivator activity retained by these two C-terminally truncated p300 mutants is likely a function of protein-protein interactions that result in recruitment of transcriptional machinery to the transcription start site (i.e., HAT-independent activities). In some promoter contexts, such HAT-independent activities may suffice to maintain normal p300 function. For example, it has been shown that HAT deletion mutants of p300 can still enhance MyoD-dependent transcription, possibly by stabilizing a ternary complex between MyoD and other coactivators [30]. Thus, it is likely that HAT-deficient p300 proteins have altered p300 activity in some settings (e.g., REL-dependent transactivation), but not in others (e.g., MyoD-dependent transactivation). Therefore, we suggest that cells expressing mutant p300 proteins are distinct from p300-null cells.

*A20* is a tumor suppressor and a target gene of NF-κB, is biallelically inactivated in approximately 30% of DLBCL, and is mutated in the SUDHL2 and RC-K8 cell lines [28,31,32]. Knockdown of p300ΔC-1087 resulted in increased expression of A20 in RC-K8 cells (Figures 4d,e). That observation and the presence of p300ΔC-1087 at the *A20* promoter (Figure 4f) suggest that p300ΔC-1087 directly reduces *A20* gene expression in RC-K8 cells, leading to reduced A20 protein. Reduced A20 protein activity appears to be essential for RC-K8 and SUDHL2 survival, as re-expression of wild-type A20 induces apoptosis in both cell types [28]. Therefore, it appears that A20 activity is reduced in SUDHL2 and RC-K8 cells by both mutation [28] and transcriptional repression mediated by mutant p300.

Knockdown of p300ΔC-1087 in RC-K8 cells also resulted in increased IκBα expression (Figures 4e,f). We have previously shown that RC-K8 cells have inactivating mutations in three of four copies of the *NFKBIA* gene, express little wild-type IκBα protein, and consequently show high levels of both nuclear REL DNA-binding activity and REL target gene expression [27]. Forced expression of wild-type IκBα protein slows the growth of RC-K8 cells, presumably due to inhibition of REL [27].

Taken together, these results suggest that C-terminally truncated p300 proteins contribute to the oncogenic state in SUDHL2 and RC-K8 cell lines, at least in part, by reducing expression of both A20 and IκBα, which allows for tolerable and optimal levels of nuclear NF-κB activity that promote cell growth. Indeed, both cell lines belong to the ABC-subtype of DLBCL, which is characterized by constitutive NF-κB activity and sensitivity to NF-κB inhibitors [33]. Overall, we propose that the high levels of nuclear REL-driven transactivation of target genes that is unleashed by mutations in the REL/NF-κB inhibitors A20 and IκBα in RC-K8 and SUDHL2 cells is tempered by expression of p300ΔC proteins, which act as muted REL coactivators. The model that moderate, chronic increases in REL-driven target gene expression are optimal for B-lymphoid cell transformation is reminiscent of the mutation-driven activation of the lymphoid cell-specific oncoprotein v-Rel, which is a chronic low level activator of target gene expression as compared to c-Rel [34].

The CH1 domain of p300 is retained in both p300ΔC-1087 and p300ΔC-820, and is required for the interaction of p300 with REL (Figure 3b). Thus, the CH1 domain and interaction with REL may be important for the growth-promoting activity of truncated p300 proteins in DLBCL. In support of this hypothesis, Kimbrel *et al*. [21] used a mouse *in vivo* reconstitution system to show that expression of a HAT domain mutant of p300 *increased* the proliferative potential of hematopoietic stem and progenitors cells, whereas expression of a CH1 domain mutant resulted in severe defects in hematopoiesis.

We have found that DLBCL cell lines with reduced expression of wild-type p300 generally have low levels of H3K14 and H3K18 acetylation (Figure 5, Additional files 2 and 3). It has been shown that p300 and CBP are able to acetylate H3K14 and H3K18 *in vitro* and that p300 and CBP are required for H3K18 acetylation *in vivo*[35,36]. Additionally, hypoacetylation of H3K18 by inhibition of p300 and CBP stimulates cell cycling in quiescent human cells and has been associated with recurrence of low-grade prostate cancer in patient studies [36-38]. Developmental studies in mice have shown that acetylation of H3K14 is associated with gene activation [39], suggesting that its reduction in RC-K8 and SUDHL2 cells prevents expression of target genes specifically related to growth inhibition and/or apoptosis. Consistent with this hypothesis, H3K14 acetylation at the promoter of the cell cycle inhibitor p21 is upregulated 10-fold in response to treatment with the topoisomerase II inhibitor doxorubicin, and is required for stress-induced cell-cycle arrest in human cancer cell lines [40]. We suggest that expression of truncated p300 and the associated reduction of wild-type p300 is one mechanism that can lead to reduced acetylation of H3K14 and H3K18, which contributes to DLBCL cell growth. Of note, SUDHL2 and RC-K8 cells are sensitive to apoptosis induced by treatment with two HDAC inhibitors [41].

Our findings contradict a previously published report on the lack of full-length p300 protein in the BJAB, SUDHL8, and Farage DLBCL cell lines [3]. We were able to show that multiple cell line stocks, including ones used in the conflicting report, express easily detectable levels of full-length p300 protein. Thus, we believe that the lack of full-length p300 protein in these three cell lines reported in Pasqualucci *et al*. [3] was due to a technical error. Northern blotting data reported by Pasqualucci *et al*. [3] further support our results, in that the BJAB, SUDHL8, and Farage cell lines express detectable levels of full-length p300 mRNA, whereas SUDHL2 cell do not. Moreover, full-length p300 protein expression in BJAB and Farage cells has been reported by several others [7,22-25].

## Conclusions

Based on our continuing studies, we propose that elimination of p300 HAT activity and expression of HAT-deletion p300 mutants both play oncogenic roles in DLBCL. Specifically, the HAT-independent activities retained in the truncated p300 proteins contribute to the proliferation and soft agar growth of certain DLBCL cell lines *in vitro*. Future studies will be aimed at identifying other pathways and genes in DLBCL cells that are affected by the expression of p300 mutants.

## Materials and methods

### Plasmids

DNA manipulations were carried out by standard methods [42]. Complete details of subclones and primers used in this study are described in supplementary information and at http://www.nf-kb.org. GST-p300-CH1 and GST-p300-N340 expression plasmids were kindly provided by Andrew Kung and have been described previously [14]. All recombinant DNA and human cell line work was conducted with BSL-2 level approval of the Boston University Institutional Biosafety Committee (approval number 11-072).

### Cell culture

A293, A293T, BOSC23 human embryonic kidney cells, DF-1 chicken fibroblasts, and RC-K8 cells were cultured in Dulbecco’s modified Eagle’s medium (DMEM) supplemented with 10% fetal bovine serum (FBS) (Biologos, Montgomery, IL, USA) as described previously [43]. BJAB cells were cultured in DMEM supplemented with 20% FBS. SUDHL2, Ramos, and Farage cells were cultured in RPMI supplemented with 10% FBS. SUDHL8 cells were cultured in RPMI supplemented with 20% FBS. The human B-lymphoma cell lines are classified as follows: DLBCL (BJAB [3,16,44], Farage [2,45], RC-K8 [8], SUDHL2 [3], SUDHL8 [3,19]), and Burkitt’s lymphoma (Ramos).

Transfection of A293, A293T, BOSC23, and DF-1 cells was performed as described previously [18]. For Western blotting and indirect immunofluorescence, cells were processed 48 h after addition of the transfection mix.

Design of control and *EP300* short hairpin RNAs (shRNA), generation of virus stocks, and infections were performed as described previously [18]. Two days after infection, SUDHL2 and RC-K8 cells were selected with 1 μg/ml puromycin (Sigma, St. Louis, MO, USA), respectively, for 2-4 weeks and maintained in puromycin throughout all experiments.

### Western blotting and indirect immunofluorescence

Whole-cell lysates were prepared in AT buffer containing protease inhibitors as described previously [46] and were analyzed by Western blotting according to standard methods [10]. High molecular weight proteins (full-length p300 and CBP) were transferred at 260 mA for 2.5 h at 4°C using a modified large-protein transfer buffer (20 mM Tris, 150 mM glycine, 0.05% SDS, 10% methanol) as described previously [10]. Western blots were quantified using ImageJ software [47].

The following antisera were used: rabbit anti-p300 (1:200; anti-N-terminal, sc-584, Santa Cruz Biotechnology, Santa Cruz, CA, USA), rabbit anti-REL (1:200; obtained from Nancy Rice [43]), mouse anti-CBP (1:200; sc-7300, Santa Cruz Biotechnology), mouse anti-A20 (1:200, 550859, BD Pharmingen, Franklin Lakes, NJ, USA), mouse anti-IκBα (1:1000, 4814, Cell Signaling Technology), and rabbit anti-β-tubulin (1:500; sc-9104, Santa Cruz Biotechnology).

Indirect immunofluorescence was performed as described previously [18,43] using anti-p300 (1:50; sc-584, Santa Cruz Biotechnology) primary antibody and fluorescein isothiocyanate (FITC)-conjugated goat anti-rabbit IgG (1:80; Sigma) secondary antibody. Nuclei were also stained with 4’,6-diamidino-2-phenylindole (DAPI). Cells were visualized using a fluorescent microscope (Olympus FLUOVIEW Laser Scanner Microscope BX 50, Center Valley, PA, USA).

### Co-immunoprecipitation

For co-immunoprecipitation of overexpressed proteins, A293 cells in 100-mm dishes were co-transfected with 10 μg pcDNA-Flag-REL and 10 μg pCMVβ-p300ΔC-820. Two days later, nuclear extracts were prepared using a Nuclear Complex Co-IP Kit (cat no. 54001, Active Motif, Carlsbad, CA, USA) according to the manufacturer’s instructions. Nuclear extracts containing 300 μg of protein were incubated with anti-p300 antiserum or rabbit pre-immune serum for 3 h at 4°C. 100 μl of a 50% slurry of Protein A Sepharose CL-4B (GE Healthcare Life Sciences, Pittsburgh, PA, USA) was added and the sample was incubated for an additional 1 h at 4°C. The beads were then washed in PBS and proteins were eluted by heating at 95°C in SDS sample buffer. Proteins were electrophoresed on a 7.5% SDS-polyacrylamide gel and transferred to a nitrocellulose membrane as described above. One percent of the amount of nuclear extract used for one immunoprecipitation (3 μg) was included on the gel as an input lane. The membranes were then subjected to anti-REL Western blotting.

Co-immunoprecipitation of endogenous REL and p300ΔC-820 in SUDHL2 cells was performed using the Nuclear Complex Co-IP Kit as described by the manufacturer (cat. no. 54001, Active Motif). Three micrograms of normal rabbit IgG (sc-2027, Santa Cruz Biotechnology) or anti-p300 antiserum (sc-584, Santa Cruz Biotechnology) was incubated with 250 μg of nuclear extract in IP Low Buffer (Active Motif) for 3 h at 4°C. 50 μl of a 50% slurry of Protein A Sepharose CL-4B (GE Healthcare Life Sciences) was added, and samples were incubated for an additional 3 h. Beads were then washed with IP Low Buffer and proteins were eluted by heating at 95°C in SDS sample buffer. Proteins were electrophoresed on a 6% SDS-polyacrylamide gel and transferred to a nitrocellulose membrane as described above. Ten percent of the amount of nuclear extract used for one immunoprecipitation (25 μg) was included on the gel as an input lane. The membranes were then subjected to anti-REL or anti-p300 Western blotting.

### GST pulldown assays

GST pulldown assays followed by Western blotting were performed as described previously [10]. One percent of the amount of extract used for each pulldown (30 μg) was included on the gel as an input lane. The membrane was then subjected to anti-p300 or anti-REL Western blotting.

### Luciferase reporter assays

Luciferase reporter assays were performed using the Luciferase Assay System (Promega, Madison, WI, USA) as described previously [18]. A293 cells in 35-mm plates were transfected with 0.5 μg of reporter plasmid pGL2-3×-κB-luciferase and 0.5 μg of normalization plasmid pRSV-βgal. Cells were co-transfected with 0.5 μg of pcDNA-REL or pcDNA3.1 vector alone, along with 0.5 μg of pCMVβ-p300, pCMVβ-p300ΔC, or vector alone. In titration experiments (Figure 2d), cells were transfected with 0.5 μg of pcDNA-REL, and increasing amounts of pCMVβ-p300, pCMVβ-p300ΔC-1087, or pCMVβ-p300ΔC-820. Increasing amounts of each p300 plasmid were titrated in until luciferase activity reached a plateau. For all luciferase reporter assay experiments, total DNA per transfection was kept constant by including varying amounts of pcDNA3.1 vector. Luciferase and β-galactosidase activities were determined, and values were normalized to the relevant vector control (1.0).

Statistical analyses were performed using a paired one-tailed t-test and p < 0.05 was considered significant.

### Cell proliferation and soft agar assays

Cell proliferation and soft agar colony assays were performed as described previously [18,48]. Equal numbers (2000 and 5000) of SUDHL2 cells expressing the indicated shRNA were placed in soft agar containing RPMI with 20% FBS and 0.3% Bacto Agar (Difco, Franklin Lakes, NJ, USA), and plates were incubated at 37°C in a humid incubator with 5% CO_2_. Macroscopic colonies were counted 14 days after plating.

### Chromatin immunoprecipitation assays and qPCR

For ChIP assays, approximately 10^8^ RC-K8 cells were fixed with 3% formaldehyde for 20 min at room temperature. Cells were then rinsed three times with ice-cold PBS and nuclear lysate was prepared as described previously [49]. Samples containing 350 μg of protein were then incubated at 4°C overnight with either rabbit anti-p300 antiserum or pre-immune serum. The next day, 50 μl of a 50% slurry of protein A beads was added and the reaction was incubated for 3 h at 4°C. Beads were washed with RIPA buffer (50 mM Tris-HCl pH 7.2, 150 mM NaCl, 1% deoxycholic acid, 1% Triton X-100, 0.1% SDS) and then TE supplemented with 50 mM NaCl. Beads were eluted in TE with 2% SDS for 15 min at 65°C. Crosslink reversal and DNA purification were performed as described previously [49]. Purified DNA was then subjected to qPCR using primers to amplify the *TNFAIP3* (*A20*) promoter. PCRs were performed in the ABI Prism 7900HT Sequence Detection System (Applied Biosystems, Foster City, CA, USA) using 40 cycles of 94°C for 15 s and 60°C for 1 min. The primers used were 5’-CAGCCCGACCCAGAGAGTCAC and 5’-TTCGTGGCGGGCCAAG [50]. Cycle threshold (C_t_) values for p300 were normalized to the pre-immune serum control values (1.0). Error bars represent standard error.

### PCR and real-time quantitative PCR

Two hundred ng of genomic DNA from SUDHL2 cells was subjected to PCR using forward and reverse primers specific for sequences surrounding exon 14 of *EP300*. The primers used were 5’- AGCATAGGCAGGCCCTAGA and 5’- TATGCTTGGGGGAGTATGGT. Sequencing of the amplified fragment was performed by Eurofins MWG Operon (Huntsville, AL, USA).

For qPCR of mRNA, total RNA was first isolated from RC-K8 cells using TRIzol Reagent (Invitrogen, Grand Island, NY, USA) according to the manufacturer’s protocol. The mRNA was reverse transcribed into cDNA using M-MLV reverse transcriptase (Promega) and random primers (Promega). One thirtieth of the synthesized cDNA was combined with gene-specific primers and Power SYBR Green PCR Master Mix (Applied Biosystems). PCRs were performed as described above. C_t_ values were obtained for each sample and normalized to C_t_ values for *GAPDH* cDNA amplification (ΔC_t_) and then to C_t_ values from control shRNA-expressing RC-K8 cells (ΔΔC_t_) using methods described previously [51]. The fold change in mRNA was normalized to the fold change in *GAPDH* mRNA expression (1.0) between p300 and control knockdown RC-K8 cells. Primers used were *A20*: 5’- CGCTCAAGGAAACAGACACA and 5’- CTTCAGGGTCACCAAGGGTA; *CCR7*: 5’-TGAGGTCACGGACGATTACAT and 5’- GTAGGCCCACGAAACAAATGAT; *NFKBIA*: 5’- CTCCGAGACTTTCGAGGAAATAC and 5’- GCCATTGTAGTTGGTAGCCTTCA; *TRAF1*: 5’- TCCTGTGGAAGATCACCAATGT and 5’- GCAGGCACAACTTGTAGCC; *TNF*: 5’- GAGGCCAAGCCCTGGTATG and 5’- CGGGCCGATTGATCTCAGC; *LTA*: 5’- CATCTACTTCGTCTACTCCCAGG and 5’-CCCCGTGGTACATCGAGTG; *A1*: 5’- TACAGGCTGGCTCAGGACTAT and 5’- CGCAACATTTTGTAGCACTCTG; *GAPDH*: 5’- TGGTATCGTGGAAGGACTCATGAC and 5’- ATGCCAGTGAGCTTCCCGTTCAGC.

Statistical analyses were performed using a paired two-tailed t-test, and p < 0.05 was considered significant.

### Quantification of histone acetylation via mass spectrometry

Cell lines were maintained in healthy conditions for several passages before histones were purified using the Active Motif Histone Purification Kit (cat no. 40025) according to the manufacturer’s instructions. Concentrations were determined using Nanodrop, 5 μg of each sample was chemically propionylated using 1.5 μl propionic anhydride, and ammonium hydroxide was used to quickly adjust the pH to approximately 8.0 [52]. Samples were then incubated at 51°C for 1 h followed by trypsin digestion overnight at 37°C. The fraction of acetylated to unmodified at a given histone H3 site was performed as described previously [53]. Means and 95% confidence intervals of acetylation values for different cell lines were calculated.

## Abbreviations

aa: Amino acid (s); bp: Base pair; CH1: Cysteine/histidine-rich 1; CI: Confidence interval; DLBCL: Diffuse large B-cell lymphoma; DMEM: Dulbecco’s modified Eagle’s medium; FBS: Fetal bovine serum; GST: Glutathione S-transferase; HAT: Histone acetyltransferase; PCR: Polymerase chain reaction; PEI: Polyethylenimine; shRNA: Short hairpin RNA; TAD: Transactivation domain.

## Competing interests

The authors declare that they have no competing interests.

## Authors’ contributions

LH performed all experiments except for full-length p300 detection in multiple DLBCL isolates (JL-P) and mass-spectrometry analysis of histone H3 acetylation (RAH, AJA). LH and TDG designed the studies and analyzed and interpreted the data. LH and TDG wrote the manuscript. All authors read and approved the final manuscript.

## Supplementary Material

Additional file 1 **SUDHL2 cells do not have a wild-type *EP300 *** **sequence at codon 821.** Genomic DNA corresponding to exon 14 of *EP300* was sequenced in SUDHL2 cells. Sequencing was performed in the forward (top strand) and reverse (bottom strand) direction. Chromatograms and corresponding nucleotides are shown in the forward (top strand) and reverse (bottom strand) direction. The reported C2856T nonsense mutation at codon 821 [3] is highlighted. Click here for file

Additional file 2 **Relative histone H3 acetylation profile in DLBCL cell lines.** Total histones were extracted from the indicated cell lines. For each H3 lysine residue, the amount of the acetylated lysine residue is reported relative to total amount of acetylated plus unmodified residue. To generate the relative fraction of acetylated H3, a single common maximum value and single common minimum value were applied to all lysine residues analyzed, and absolute values (Figure 5) within each residue were distributed proportionally across this normalized range. The relative fraction of acetylated H3 for all cell lines is shown as a box plot for each lysine residue. Cell lines indicated by red diamonds express undetectable levels of wild-type p300 (RC-K8, SUDHL2) or wild-type CBP (Karpas422) by Western blotting. Cell lines indicated by blue circles express detectable levels of full-length p300 and CBP by Western blotting. Click here for file

Additional file 3 **Fraction of acetylated lysine residue relative to total acetylated plus unmodified residue in Histone H3.** Fraction of acetylated lysine residue as determined by mass spectrometry for B-lymphoma cell lines. Values are plotted in Figure 5. Click here for file
