# Supplementary material for: Histone acetyltransferase-deficient p300 mutants in diffuse large B cell lymphoma have altered transcriptional regulatory activities and are required for optimal cell growth
Source: Mol Cancer. 2014 Feb 15;13:29. doi: 10.1186/1476-4598-13-29 (PMC3930761; doi:10.1186/1476-4598-13-29)
Supplement: Additional file 1 — SUDHL2 cells do not have a wild-type EP300 sequence at codon 821. Genomic DNA corresponding to exon 14 of EP300 was sequenced in SUDHL2 cells. Sequencing was performed in the forward (top strand) and reverse (bottom strand) direction. Chromatograms and corresponding nucleotides are shown in the forward (top strand) and reverse (bottom strand) direction. The reported C2856T nonsense mutation at codon 821 [3] is highlighted. [file 1476-4598-13-29-S1.pdf]

Additional File 1

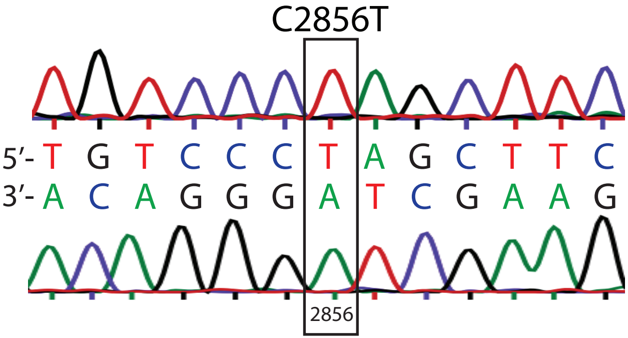

SUDHL2 cells do not have a wild-type EP300 sequence at codon 821.
Genomic DNA corresponding to exon 14 of EP300 was sequenced in SUDHL2 cells. Sequencing was performed in the forward (top strand) and reverse (bottom strand) direction. Chromatograms and corresponding nucleotides are shown in the forward (top strand) and reverse (bottom strand) direction. The reported C2856T nonsense mutation at codon 821 [3] is highlighted.
