# Supplementary material for: Histone acetyltransferase-deficient p300 mutants in diffuse large B cell lymphoma have altered transcriptional regulatory activities and are required for optimal cell growth
Source: Mol Cancer. 2014 Feb 15;13:29. doi: 10.1186/1476-4598-13-29 (PMC3930761; doi:10.1186/1476-4598-13-29)
Supplement: Additional file 2 — Relative histone H3 acetylation profile in DLBCL cell lines. Total histones were extracted from the indicated cell lines. For each H3 lysine residue, the amount of the acetylated lysine residue is reported relative to total amount of acetylated plus unmodified residue. To generate the relative fraction of acetylated H3, a single common maximum value and single common minimum value were applied to all lysine residues analyzed, and absolute values (Figure 5) within each residue were distributed proportionally across this normalized range. The relative fraction of acetylated H3 for all cell lines is shown as a box plot for each lysine residue. Cell lines indicated by red diamonds express undetectable levels of wild-type p300 (RC-K8, SUDHL2) or wild-type CBP (Karpas422) by Western blotting. Cell lines indicated by blue circles express detectable levels of full-length p300 and CBP by Western blotting. [file 1476-4598-13-29-S2.pdf]

Additional File 2

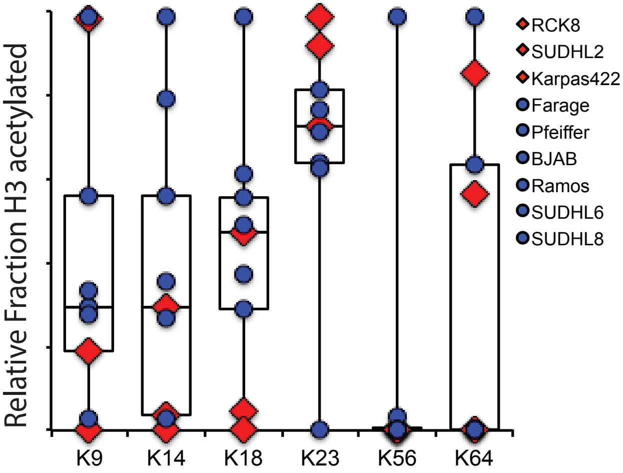

Relative histone H3 acetylation profile in DLBCL cell lines.

Total histones were extracted from the indicated cell lines. For each H3 lysine residue, the amount of the acetylated lysine residue is reported relative to total amount of acetylated plus unmodified residue. To generate the relative fraction of acetylated H3, a single common maximum value and single common minimum value were applied to all lysine residues analyzed, and absolute values (Figure 5) within each residue were distributed proportionally across this normalized range. The relative fraction of acetylated H3 for all cell lines is shown as a box plot for each lysine residue. Cell lines indicated by red diamonds express undetectable levels of wild-type p300 (RC-K8, SUDHL2) or wild-type CBP (Karpas422) by Western blotting. Cell lines indicated by blue circles express detectable levels of p300 and CBP by Western blotting.
