# Supplementary material for: Histone acetyltransferase-deficient p300 mutants in diffuse large B cell lymphoma have altered transcriptional regulatory activities and are required for optimal cell growth
Source: Mol Cancer. 2014 Feb 15;13:29. doi: 10.1186/1476-4598-13-29 (PMC3930761; doi:10.1186/1476-4598-13-29)
Supplement: Additional file 3 — Fraction of acetylated lysine residue relative to total acetylated plus unmodified residue in Histone H3. Fraction of acetylated lysine residue as determined by mass spectrometry for B-lymphoma cell lines. Values are plotted in Figure 5. [file 1476-4598-13-29-S3.pdf]

Additional File 3 Fraction of acetylated lysine residue relative to total acetylated plus unmodified residue Fraction of acetylated lysine residue relative to total acetylated plus unmodified residue

Fraction of acetylated lysine residue as determined by mass spectrometry for B-lymphoma cell lines. Values are plotted in Figure 5.

Cell line	Ratio of acetylated to acetylated plus unmodified peptides					
	K9	K14	K18	K23	K56	K64
**BJAB**	0.0004	0.1800	0.0273	0.1883	0.0000	0.0000
**Farage**	0.0038	0.2819	0.0333	0.1693	0.0012	0.0000
**Karpas422**	0.0024	0.0027	0.0035	0.1922	0.0001	0.0000
**Pfeiffer**	0.0071	0.0175	0.0175	0.2020	0.0369	0.0000
**Ramos**	0.0042	0.4967	0.0215	0.2148	0.0001	0.0000
**RC-K8**	0.0001	0.1507	0.0057	0.2417	0.0000	0.0000
**SUDHL2**	0.0124	0.0214	0.0265	0.2596	0.0001	0.0000
**SUDHL6**	0.0124	0.3977	0.0305	0.0061	0.0000	0.0000
**SUDHL8**	0.0035	0.1372	0.0515	0.1663	0.0001	0.0000
